# Multiple Complexes of Nitrogen Assimilatory Enzymes in Spinach Chloroplasts: Possible Mechanisms for the Regulation of Enzyme Function

**DOI:** 10.1371/journal.pone.0108965

**Published:** 2014-10-01

**Authors:** Yoko Kimata-Ariga, Toshiharu Hase

**Affiliations:** Institute for Protein Research, Osaka University, Suita, Osaka, Japan; Mount Allison University, Canada

## Abstract

Assimilation of nitrogen is an essential biological process for plant growth and productivity. Here we show that three chloroplast enzymes involved in nitrogen assimilation, glutamate synthase (GOGAT), nitrite reductase (NiR) and glutamine synthetase (GS), separately assemble into distinct protein complexes in spinach chloroplasts, as analyzed by western blots under blue native electrophoresis (BN-PAGE). GOGAT and NiR were present not only as monomers, but also as novel complexes with a discrete size (730 kDa) and multiple sizes (>120 kDa), respectively, in the stromal fraction of chloroplasts. These complexes showed the same mobility as each monomer on two-dimensional (2D) SDS-PAGE after BN-PAGE. The 730 kDa complex containing GOGAT dissociated into monomers, and multiple complexes of NiR reversibly converted into monomers, in response to the changes in the pH of the stromal solvent. On the other hand, the bands detected by anti-GS antibody were present not only in stroma as a conventional decameric holoenzyme complex of 420 kDa, but also in thylakoids as a novel complex of 560 kDa. The polypeptide in the 560 kDa complex showed slower mobility than that of the 420 kDa complex on the 2D SDS-PAGE, implying the assembly of distinct GS isoforms or a post-translational modification of the same GS protein. The function of these multiple complexes was evaluated by in-gel GS activity under native conditions and by the binding ability of NiR and GOGAT with their physiological electron donor, ferredoxin. The results indicate that these multiplicities in size and localization of the three nitrogen assimilatory enzymes may be involved in the physiological regulation of their enzyme function, in a similar way as recently described cases of carbon assimilatory enzymes.

## Introduction

Intracellular enzymes are pertinently distributed and/or co-localized with functionally related proteins rather than evenly dispersed within cells or organelles, and dynamically change their states in response to environmental changes, for the biological reactions to proceed efficiently in a highly controlled fashion [1], [2]. Various chloroplast enzymes are subjected to light/dark modulation of their activity through redox modulation [3], whose rate is adjusted by other factors such as specific metabolites [4]. In some cases, the reversible changes in the redox and activation states are accompanied by oligomerization and re-dissociation of transient complexes. For the enzymes of photosynthetic carbon assimilation, several lines of evidence have suggested that enzymes of the Calvin cycle associate to form multiprotein complexes. A multiprotein complex including two major Calvin cycle enzymes and a small protein (CP12) has been identified [5], and its reversible dissociation allowing for rapid regulation of enzyme activity was shown to be mediated by thioredoxin in response to changes in light availability [6]. Another recent finding for the key photosynthetic enzyme, ferredoxin-NADP^+^ oxidoreductase (FNR), is its reversible association with thylakoid binding proteins (Tic 62 and TROL) in response to light signal and stromal pH, which regulates the stability and dynamic light-dependent membrane tethering of FNR [7], [8]. Assimilation of nitrate is another major biological process in photosynthetic organisms, and has a large effect on plant growth and development [9]. Nitrate transported into cells is reduced to nitrite by nitrate reductase present in the cytosol and is further reduced to ammonium by nitrite reductase (NiR) in the chloroplast. The resulting ammonium is fixed as the amine group of Gln by glutamine synthetase (GS), and then two molecules of Glu are synthesized from Gln and 2-oxoglutarate by glutamate synthase (also called as glutamine oxoglutarate aminotransferase; GOGAT). The holoenzyme of NiR contains a siroheme and a 4Fe-4S cluster, and the GOGAT holoenzyme contains an FMN and a 3Fe-4S cluster. NiR, GS and GOGAT are nuclear-encoded and known to be located in the stromal fraction of chloroplasts in higher plants. NiR and GOGAT require reducing powers for their reactions, and their physiological electron donor is ferredoxin (Fd) (or NADH in the case of GOGAT), which is reduced by light-dependent reactions of the photosynthetic electron-transfer chain. These enzymes are known to be highly regulated during development and by external conditions such as light availability and nitrogen availability at the level of protein expression (reviewed in [9], [10]). Findings of the multiprotein complexes of the Calvin cycle enzymes and FNR suggest that similar post-translational mechanisms may also exist for the regulation of nitrogen assimilatory enzymes. A recent study of protein profiles of GS gene products in the legume *Medicago truncatula* showed that the GS polypeptides assembled into organ-specific protein complexes with different molecular mass, implying organ-specific post-translational modifications under defined physiological conditions [11]. In this study, the possibility for the formation of protein complexes involving three nitrogen assimilatory enzymes, NiR, GS and GOGAT, was investigated using spinach leaves, by the method of blue native PAGE (BN-PAGE). BN-PAGE is a powerful tool for separating protein complexes from biological membranes and the soluble fraction under native conditions [12], which was also used for the above analyses of the Calvin cycle enzymes [6] and FNR [7], [8]. In spinach, NiR is encoded in a single gene per haploid genome, and expressed as a single polypeptide of 65 kDa protein, whose X-ray crystal structure was recently solved [13]. GOGAT in plant plastids is present as two distinct classes, with different physiological electron donors, Fd and NADH. Fd-dependent GOGAT (Fd-GOGAT) is encoded in a single gene in spinach, expressed as a large single polypeptide of 165 kDa, and accounts for more than 95% of the total GOGAT activity in photosynthetic plant tissue [14]. NADH-GOGAT in plants is present as a 240 kDa polypeptide containing an additional NADH-binding region at the C-terminus. GS in higher plants also exists as two distinct types, cytosolic GS (GS1) and plastidic GS (GS2), with different polypeptide sizes of 38–40 kDa and 42–45 kDa, respectively [15]. Plant GS1 and GS2 polypeptides assemble into decameric complexes to be active holoenzymes [16],[17]. In this study, we found each of NiR, GS and GOGAT assembled into discrete protein complexes in the chloroplasts of spinach leaves, and the dissociation profile of these complexes varied in response to the changes in the stromal conditions. The function of these complexes was evaluated, thus suggesting the involvement of the multiplicities in size and localization of these nitrogen assimilatory enzymes in the physiological regulation of enzyme function, in a similar way as the cases of the carbon assimilatory enzymes.

## Materials and Methods

### Isolation of stroma and thylakoid proteins

Chloroplasts were prepared essentially as described by Mach [18] from mature leaves of spinach (*Spinacia oleracea*) purchased at a local market, and then ruptured in 20 mM Hepes-KOH, pH 7.5 by repeated freeze-thawing at −20°C, unless otherwise specified. Following centrifugation at 10,000×g for 5 min at 4°C, the supernatant was filtered through a 0.2-µm filter membrane, and reserved as a stromal fraction. The pellet was re-suspended in the same buffer and centrifuged as above; this washing step was repeated twice in order to remove residual stromal components. The resulting pellet of the membrane fraction was re-suspended for solubilization in a Native PAGE sample buffer (50 mM Bis-Tris-HCl, pH 7.2, 50 mM NaCl and 10% glycerol) (life technologies) containing 1% n-dodecyl-ß-D-maltoside (DDM), at a chlorophyll concentration of 0.5 mg/ml, and then incubated for 30 min at 4°C with constant rotation, followed by centrifugation at 20,000×g for 15 min at 4°C. The supernatant was reserved as a fraction of thylakoid proteins (possibly containing proteins from other plastid membranes such as envelop), adjusted to 0.25% Coomassie brilliant blue (CBB) G-250, and used for BN-PAGE analysis. For the preparation of proteins from whole chloroplasts, isolated chloroplasts were directly suspended in the Native PAGE sample buffer containing 1% DDM, and the solubilized proteins were recovered by the same procedure as the membrane fraction described above.

### BN-PAGE, Native PAGE, two-dimensional SDS-PAGE and western blot analysis

BN-PAGE was performed at 4°C, by using a 4–16% linear polyacrylamide gradient of Native PAGE Novex Bis-Tris Gel system (life technologies) according to the manufacture's instruction. Native PAGE was performed according to the same protocol as BN-PAGE except that CBB was omitted from the running buffer and the sample loading buffer. Unless otherwise specified, samples loaded on the gel were derived from 10 µg on a chlorophyll basis of the chloroplasts whose proteins were extracted. For two-dimensional SDS-PAGE after BN-PAGE (2D BN/SDS-PAGE), the lanes were cut out after the run, and incubated in a denaturing solution composed of 1% SDS and 1% 2-mercaptoethanol for 15 min before applying on top of a 12% SDS-PAGE gel. For western blot analysis, proteins on the gels were transferred to an immobilon PVDF membrane (Millipore) and probed with polyclonal antibodies raised against maize recombinant proteins (Fd-GOGAT, GS1 and FNR) and spinach NiR; proteins were detected by using horseradish peroxidase conjugated to Protein A (life technologies) with enhanced chemiluminescence (Western ECL substrate, Bio-Rad).

### pH treatment of chloroplasts or stromal fraction

For the preparation of stroma and thylakoid proteins under different pH conditions, chloroplasts were suspended in 20 mM potassium phosphate buffer at pH 6.0 or pH 8.0, followed by repeated freeze-thawing, and incubated on ice for 30 min before the isolation of the stroma and thylakoid proteins. For the pH shift assay of stromal proteins, the isolated stromal fraction prepared by using 20 mM Hepes-KOH, pH 7.5 buffer was diluted with three-times volume of the same buffer, or 20 mM potassium phosphate buffer at pH 6.0 or pH 8.0, and incubated for 30 min at room temperature before BN-PAGE analysis. For the pH shift assay of whole chloroplasts, chloroplasts disrupted in 20 mM Hepes-KOH, pH 7.5 by repeated freeze-thawing were diluted with three-times volume of the same buffer, or 20 mM potassium phosphate buffer at pH 6.0 or pH 8.0, and incubated for 30 min at room temperature before extraction of stromal proteins for the analysis.

### DTT treatment of thylakoid membranes

Thylakoid membranes prepared by using 20 mM potassium phosphate buffer at pH 6 was solubilized in the Native PAGE sample buffer containing 1% DDM for 30 min, and then incubated for 15 min in the absence or presence of DTT at 2, 10 and 50 mM at 4°C with constant rotation. After further incubation for 15 min at room temperature, the supernatant was recovered by centrifugation at 20,000×g for 15 min at 4°C, and used for BN-PAGE and 2D BN/SDS-PAGE analyses.

### GS activity assays in gel and in solution

GS enzyme activity assays in gel and in solution were performed essentially as described by Seabra et al. [11]. For an in-gel GS activity assay, Native PAGE was performed as described above in this section, and the gel was incubated in GS activity solution and then in a stop solution containing FeCl_3_ for chelating the product [11], allowing the detection of reddish brown color corresponding to the activity. GS activity in solution was determined by quantification of γ-glutamyl hydroxamate produced by the transferase reaction of glutamine and hydroxylamine, per minute per mg of total protein [11].

### Fd-binding assay

Fd-affinity resin was prepared by using recombinant Fd from *Leptolyngbya boryana* [19] and CNBr-activated Sepharose 4B (GE Healthcare), following the manufacture's directions. For a Fd-binding assay, stromal proteins prepared by using 20 mM potassium phosphate buffer at pH 6.0 or pH 8.0 were mixed with the Fd resin, and the unbound fraction was recovered by centrifugation. The resulting resin was washed with each potassium phosphate buffer (at pH 6.0 or pH 8.0), and the bound proteins were eluted with the same buffer containing 0.2 M NaCl and analyzed by western blots after BN-PAGE.

## Results and Discussion

### Multiple complexes of nitrogen assimilatory enzymes in spinach chloroplasts

**Figure 1:**
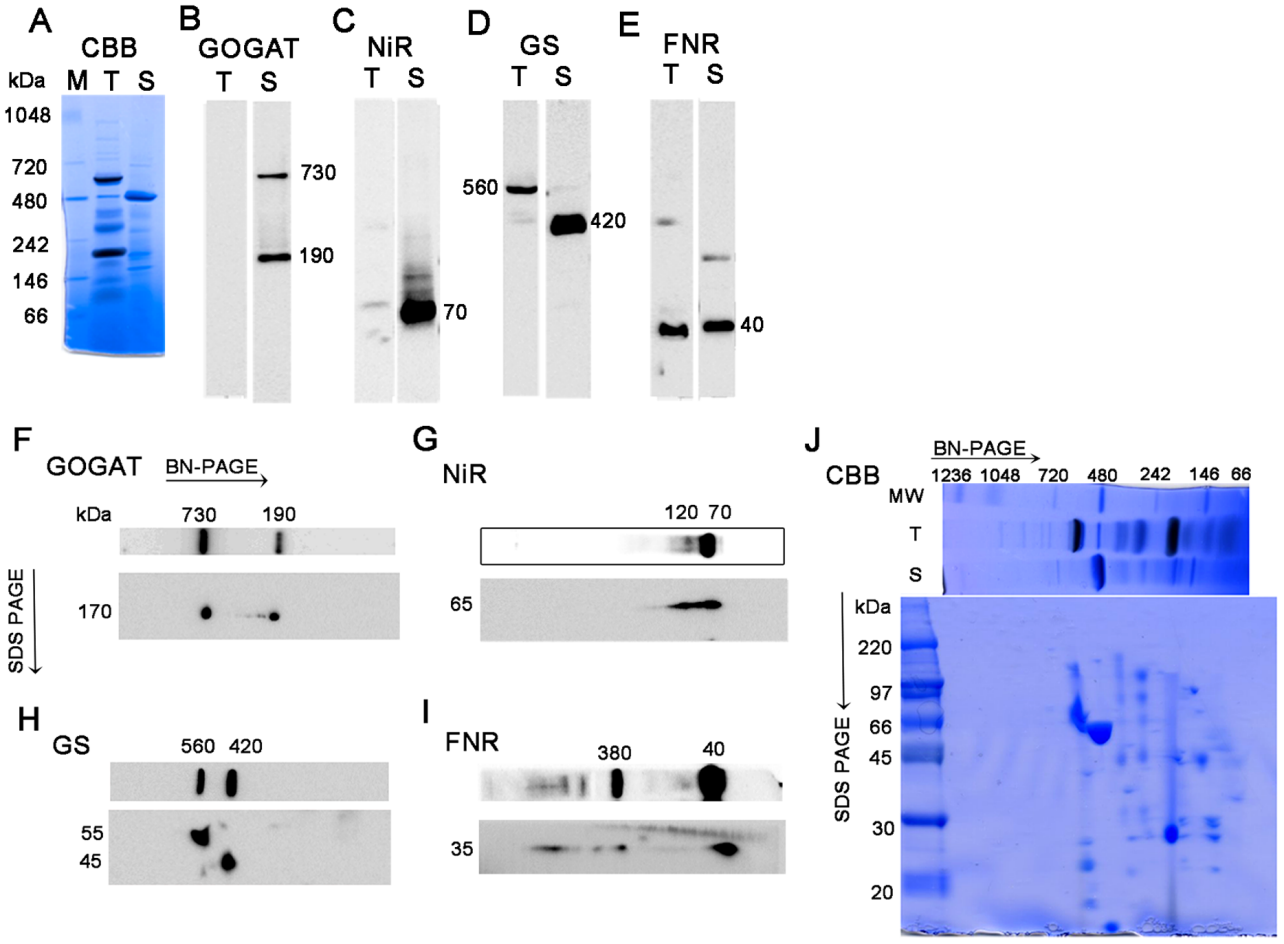
Protein complexes in spinach chloroplasts analyzed by blue-native PAGE (BN-PAGE) and SDS-PAGE. Upper panel; CBB-staining (A) and western blots (B–E) of BN-PAGE analysis of thylakoid (T) and stroma (S) proteins extracted from spinach chloroplasts. Lower panel; western blots (F–I) and CBB-staining (J) of 2D SDS-PAGE after separation by BN-PAGE of stromal proteins (F and G) and whole chloroplast proteins (H, I and 2D SDS-PAGE of J). Western blots were probed with polyclonal antibodies against each protein indicated. The numbers beside each band stand for the estimated molecular weights. All the samples loaded were derived from 10 µg on a chlorophyll basis of chloroplasts whose proteins were extracted.

Using stroma and thylakoid proteins isolated from the chloroplasts of mature spinach leaves, native protein profiles of GOGAT, NiR and GS were analyzed by western blots under BN-PAGE (Fig. 1B-D). Proteins from the two chloroplast fractions were well-separated as seen in their CBB-stained patterns (Fig. 1A). Multiple bands with various patterns among the three enzymes were detected as follows. In the analysis of GOGAT (Fig. 1B) using antibody which specifically recognizes Fd-GOGAT, two bands with different mobilities were detected exclusively in the stromal fraction (lane S). The faster-migrating band corresponds to the size of the holoform of Fd-GOGAT (approx. 190 kDa), and the size of the other band was estimated to be 730 kDa, which is about four times of the holoform. NiR is also present almost exclusively in stroma (Fig. 1C), and in addition to the major band at around 70 kDa of the holoenzyme, smear and faint bands at around 120, 170 and 340 kDa were observed. On the other hand, in GS (Fig. 1D), besides the band at 420 kDa of the decameric holoenzyme in stroma, a slower-migrating band at 560 kDa was detected in the thylakoid fraction. This 560 kDa protein was relatively resistant against the elution from thylakoid membranes under the condition of 0.1% DDM by which complexes of FNR with Tic62/TROL were mostly eluted (our preliminary experiment), indicating that its binding to thylakoid membranes is relatively tight under the current experimental conditions. In the analysis of FNR as a control (Fig. 1E), monomer size of the holoenzyme at 40 kDa was detected in both fractions, and additional bands around 400 kDa in thylakoids and 220 kDa in stroma are thought to be the complexes of FNR with Tic 62, Trol or some other proteins [7], [8]. Next, the identities of the multiple bands observed for each enzyme were analyzed by western blots under 2D BN/SDS-PAGE (Fig. 1F–I) as follows, and their physiological significances were further analyzed.

### Analysis of GOGAT complex

**Figure 2:**
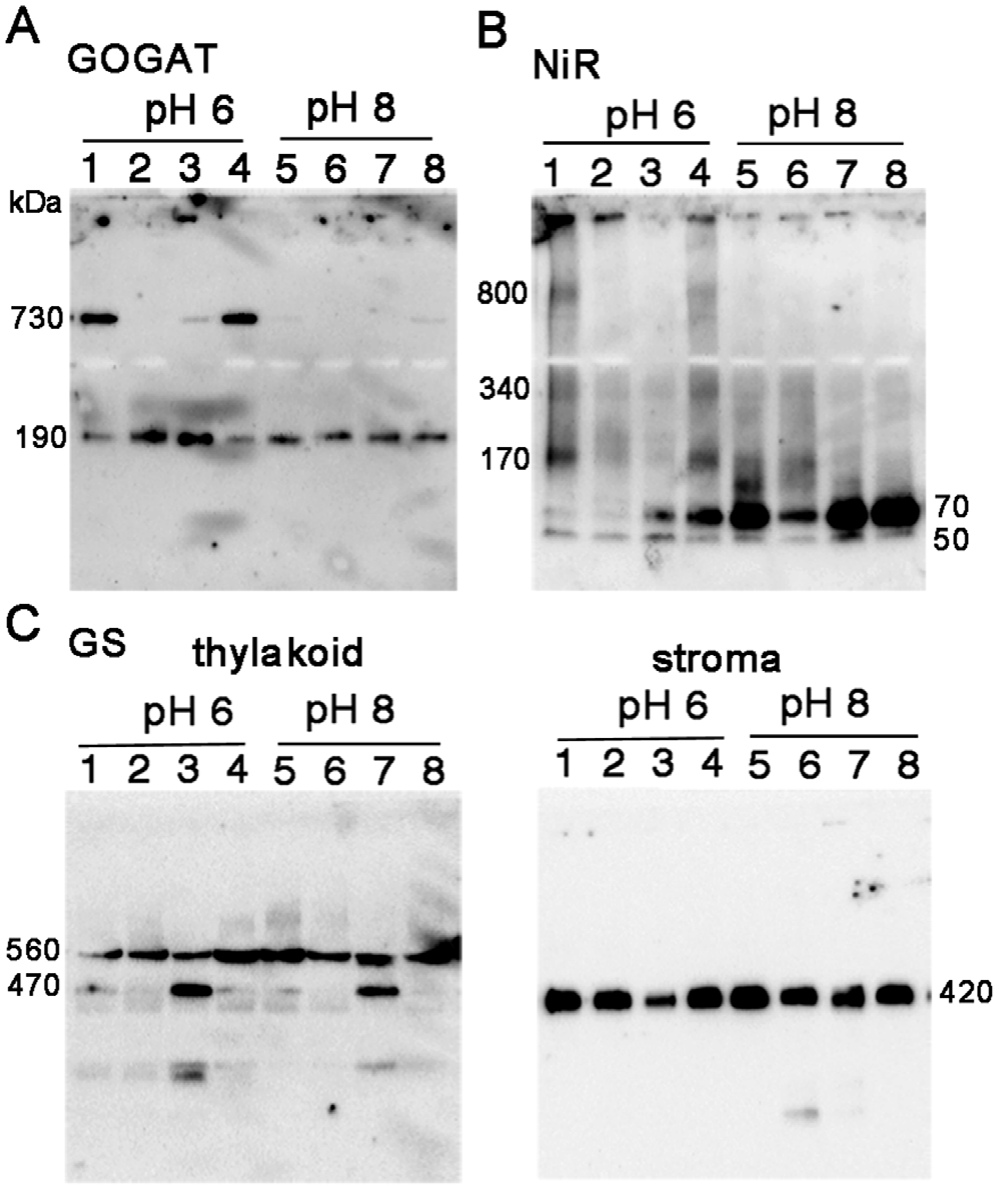
Protein distribution in response to the changes in solvent conditions of chloroplasts. Stroma and thylakoid proteins extracted from spinach chloroplasts under different solvent conditions of potassium phosphate buffer at pH 6 (lane 1) or pH 8 (lane 5) containing 0.1 M NaCl (lanes 2 and 6), 10 mM DTT (lanes 3 and 7) or 10 mM ascorbic acid (lanes 4 and 8) were analyzed by western blots after BN-PAGE. Results of stromal proteins for GOGAT (A) and NiR (B), and stroma and thylakoid proteins for GS (C) were presented. The numbers beside each band stand for the estimated molecular weights.

**Figure 3:**
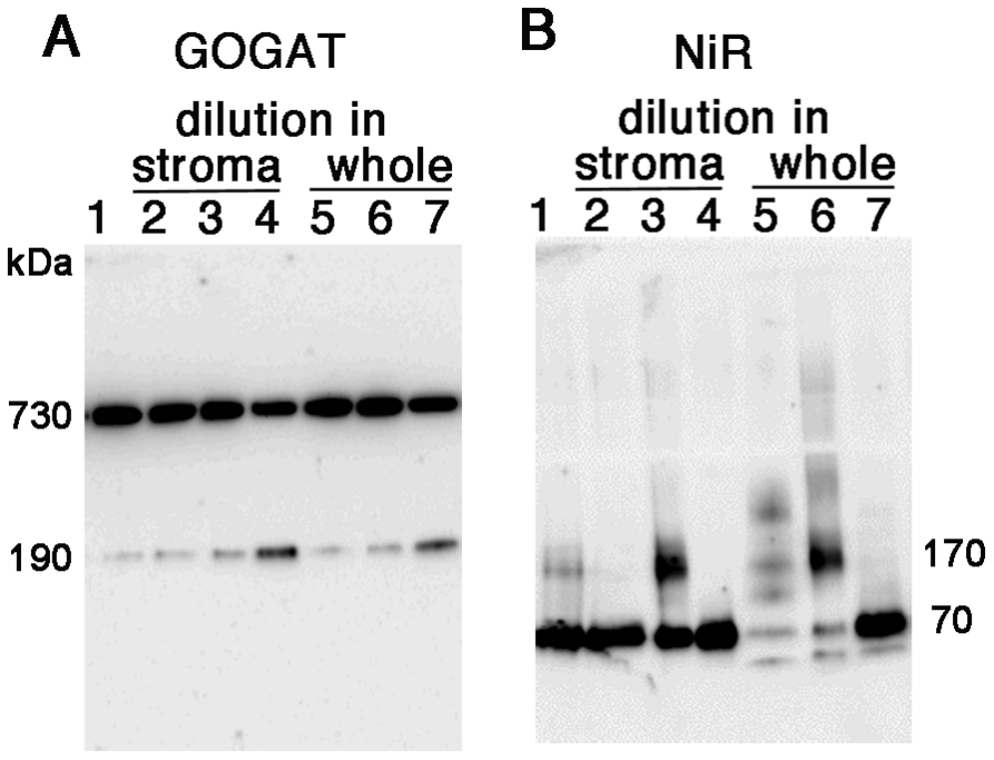
Protein distribution in response to the pH shift of stroma and whole chloroplasts. Stromal proteins extracted by using Hepes-KOH buffer at pH 7.5 (lane 1) were four-fold diluted with the same buffer (lane 2), potassium phosphate buffer at pH 6 (lane 3) or pH 8 (lane 4), incubated for 30 minutes at room temperature and analyzed by western blots for GOGAT (A) and NiR (B) after BN-PAGE. Stromal proteins extracted from whole chloroplasts treated with above three conditions were also analyzed (whole; lanes 5–7).

As shown in Figure 1F of GOGAT analysis, the two bands detected by BN-PAGE analysis exhibited the same mobility on the 2D SDS-PAGE, showing a polypeptide size of 170 kDa. Together with the previous reports that Fd-GOGAT is a single copy gene in spinach, and that NADH-GOGAT shows a very different molecular weight of 240 kDa, the 730 kDa protein was shown to be either a homomultimer (most likely a tetramer) of 190 kDa holoenzyme or its complex with other protein(s) in chloroplasts. In order to further investigate the identity and physiological significance of the 730 kDa complex, its response to the changes in the conditions of stromal solvent was analyzed (Fig. 2A). Stromal proteins were extracted from isolated chloroplasts using either a slightly acidified solvent (at pH 6) to mimic the nightly stromal environment or a slightly alkalized solvent (at pH 8) simulating the changes in the stromal pH following illumination. As shown in Figure 2A, distribution of the two bands is clearly different between the two solvent conditions (lanes 1 and 5); the 730 kDa is the major band at pH 6, on the other hand at pH 8, it mostly disappeared, and the intensity of the 190 kDa band was somewhat increased. No GOGAT signal was detected in the thylakoid fraction. This suggests that the 730 kDa complex is converted into the 190 kDa holoenzyme, and the latter form may be relatively unstable at pH 8 under the current experimental conditions. By addition of NaCl at 0.1 M (lane 2) or DTT at 10 mM (lane 3) into the stromal solvent at pH 6, the 730 kDa complex was mostly converted into the 190 kDa protein, suggesting the involvement of electrostatic interactions and disulfide bonds for the assembly of the complex. Addition of ascorbic acid at 10 mM (lane 4), which was shown to affect the redox regulation of FNR-Tic62 complex [20], had no significant effect under the current conditions. Next, whether the pH-dependent conversion between the two forms (730 kDa and 190 kDa) of GOGAT is reversible or not was investigated (Fig. 3). Stromal proteins extracted by using a standard extraction buffer at pH 7.5 (lane 1) were diluted by a buffer at pH 6 or 8 (lanes 3 and 4). Treatment with pH 8 buffer increased the 190 kDa monomer and decreased the 730 kDa complex (lane 4), while no significant change was seen by the treatment with pH 6 buffer (lane 3), in other words, re-association was not observed. Basically the same results were obtained by the same treatments of whole chloroplasts (lanes 5–7) instead of the stromal fraction, indicating that the effect of thylakoid components for the conversion is negligible. Thus, Fd-GOGAT forms a dissociable 730 kDa complex (probably a tetramer). In this regard, there is a report that bacterial NADPH-GOGAT, which consists of two subunits (α and β) and shares considerable homology with Fd-GOGAT throughout its sequence of α subunit, appears to be a (αβ)_4_ tetramer in an analytical gel filtration experiment [21] and by X-ray small angle scattering measurements [22]. Although Fd-GOGAT is generally considered to be monomeric in solution, both NADPH-GOGAT α subunit and Fd-GOGAT form similar dimers in crystals [23], [24]. Therefore, a tetramer of Fd-GOGAT may be present under physiological conditions. This 730 kDa complex dissociates into the 190 kDa monomer enzyme in response to DTT and alkalized pH (Figs. 2&3), suggesting the involvement in the redox regulation of the function of GOGAT, which will be addressed in the latter section.

### Analysis of NiR complex

The situation of NiR turned out to be partly similar to that of GOGAT. In the western analysis of 2D BN/SDS-PAGE (Fig. 1G), the multiple bands observed by BN-PAGE analysis showed the same mobility as the monomer (65 kDa), indicating that they are derived from the same NiR gene product, known to be a single copy in spinach. Therefore, the multiple bands other than the monomer NiR detected on BN-PAGE analysis were either homo-multimeric forms of NiR enzyme or its complexes with other components in the chloroplasts. Responses to the different conditions in the stromal solvent such as salt and pH (Fig. 2B) have some common features with those of GOGAT, but there also are a few intriguing differences. As in the case of GOGAT in Figure 2A, protein distributions are largely different between the treatments of pH 6 and pH 8 (lanes 1 and 5). Intensive shifts of the bands toward higher molecular weight up to around 800 kDa were observed at pH 6 (lane 1); monomer band at around 70 kDa became very faint, and instead, most abundant band of 170 kDa and less abundant, smear bands at around 340 kDa and 800 kDa are detected. The identity of the 50 kDa band is not clear but may be either a partial degradation product of NiR or a non-specifically detected band. Addition of DTT at 10 mM reduced the above shift and increased the 70 kDa monomer at both pH 6 and 8 (lanes 3 and 7), which is similar to the case of GOGAT. Unlike GOGAT, addition of NaCl at 0.1 M largely reduced the overall signals (lanes 2 and 6), and addition of ascorbic acid at 10 mM increased the monomer protein (lanes 4 and 8), implying that these agents affect the stability and/or dissociation of NiR. Another difference from GOGAT is the apparent reversible feature between NiR monomer and multiple complexes in response to the pH shifts (Fig. 3B); dilution of the stromal extract with pH 6 buffer increased the higher molecular-weight complexes, especially the 170 kDa band (lane 3 as compared to lane 1), while dilution with pH 8 buffer almost diminished the signals for those complexes (lane 4). Dilution of whole chloroplast suspension instead of the stromal extract conferred basically the same results (lanes 5–7) except that more intensive shifts of the bands were seen at lower pH, as observed in Figure 2B. Thus the conversion between monomer NiR and the higher molecular-weight complexes upon the pH shifts appears to be reversible, and their distribution varies in response to the relatively moderate changes in the solvent conditions, which may be related to the regulation of physiological function of NiR, addressed in the latter section.

### Analysis of GS complex

Another intriguing feature implying a regulatory mechanism of enzyme function was found by the analysis of the GS complex. Unlike the cases of GOGAT and NiR, the 2D SDS-PAGE analysis of the 420 kDa and 560 kDa proteins (Fig. 1H) exhibited polypeptides of different molecular weights (around 45 and 55 kDa, respectively), suggesting either that they are derived from different GS isoproteins or that a post-translational modification of the GS polypeptide occurred. The 45 kDa polypeptide corresponds to the size of plastidic GS2 protein, but the 55 kDa is rather large for the conventional plant GS proteins. In this connection, Seabra et al. [11] reported that some of the organ-specific complexes of *M. truncatula* GS2 protein, detected by western blots after Native PAGE, appeared to contain a polypeptide of higher molecular mass (50 kDa) in addition to a 42 kDa conventional GS2 polypeptide as analyzed by western blots after SDS-PAGE. Because basically only one GS2 isoprotein was shown to be expressed in this plant except in seeds, this additional polypeptide (50 kDa) was indicated to represent a post-translational modification of 42 kDa *M. truncatula* GS2 protein. Since only one GS2 isoprotein has been reported in spinach (*S. oleracea*), it is not clear whether multiple GS2 isoproteins are expressed in spinach leaves or not. Concerning membrane-bound GS proteins, a slower migrating form of the GS2 protein was shown to accumulate in the fraction of plastid membranes in root nodules of *M. truncatula* [25], and the interaction of cytosolic GS1 protein with a symbiosome membrane protein (nodulin 26) in soybean root nodules was reported recently [26].

**Figure 4:**
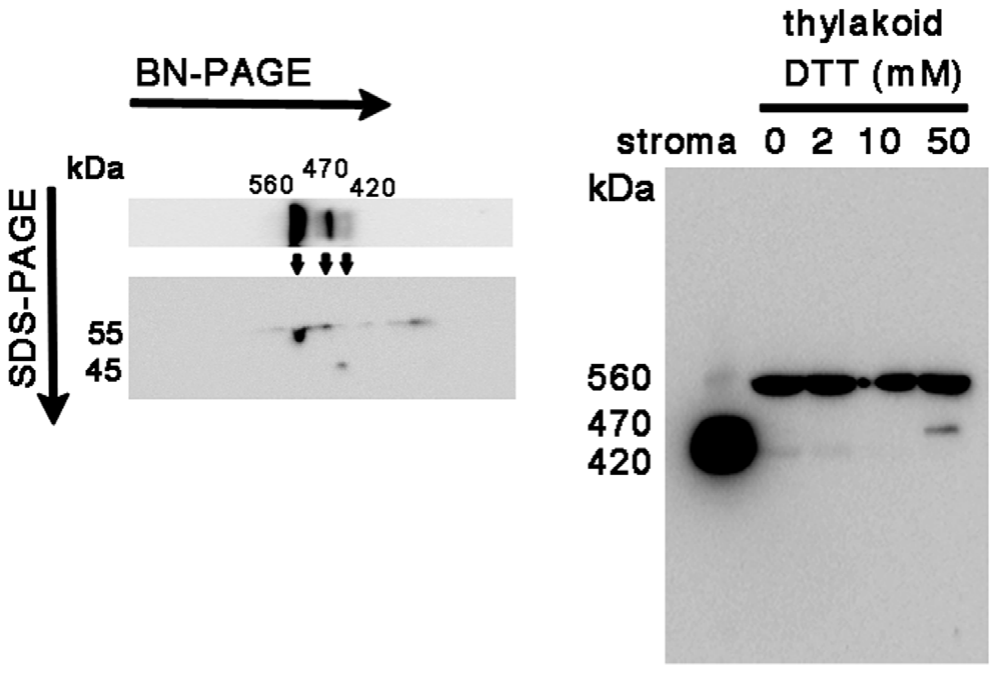
Western analysis of GS distribution in thylakoid proteins treated with DTT. Thylakoid proteins treated with DTT were analyzed by western blots of BN-PAGE (right) and 2D SDS-PAGE after BN-PAGE (left) using the GS polyclonal antibody. In the pattern of 2D SDS-PAGE (left), a faint spot at about 45 kDa is probably derived from the contamination of the stromal GS holoenzyme which is fairly abundant in the stromal fraction.

In contrast to the cases of GOGAT and NiR, no significant differences in the GS profile were observed between the two pH conditions in both the stroma and thylakoid fractions (Fig. 2C). Instead, addition of DTT produced an additional 470 kDa band below the 560 kDa band in thylakoids (lanes 3 and 7 in the left panel), together with the decrease in the amount of the stromal 420 kDa holoenzyme especially at pH 6 (lane 3 in the right panel). These results suggest either that the stromal decameric GS (420 kDa) may be recruited into thylakoid membranes under the conditions at 10 mM DTT via anchoring protein(s), or that the 470 kDa protein may be a cleaved or dissociated product of the 560 kDa protein complex. In order to seek out the identity of this 470 kDa band, 2D BN/SDS-PAGE analysis of thylakoid proteins treated with DTT was performed (left panel in Fig. 4). The 470 kDa protein on BN-PAGE exhibited the same mobility as that of the 560 kDa protein, suggesting that the 470 kDa band is probably derived from the 560 kDa complex although the subunit composition and integrity of the two complexes is not clear. Our preliminary experiment shows that the 470 kDa protein is more easily released from thylakoid membranes compared to the 560 kDa protein by the mild extraction with 0.1% DDM. This 470 kDa protein may be an intermediate complex to be released into stroma as a decameric holoenzyme by unknown mechanism. The identities of these higher molecular-weight forms are not clear, but in the root nodules of *M. truncatula*, the slower migrating form of GS2 protein as described above was shown to localize in the fraction of the plastid membrane as a catalytically inactive form [25]. Also, among the multiple GS complexes (420∼620 kDa) observed in the leaves of the same plant [11], only the lowest 420 kDa band was detected by an in-gel GS activity assay, implying the involvement of this multiplicity in the regulatory mechanism for GS enzyme activity. Thus, the activity of the GS complex in spinach chloroplasts was addressed in the following section.

### Native PAGE analysis and GS activity assays

**Figure 5:**
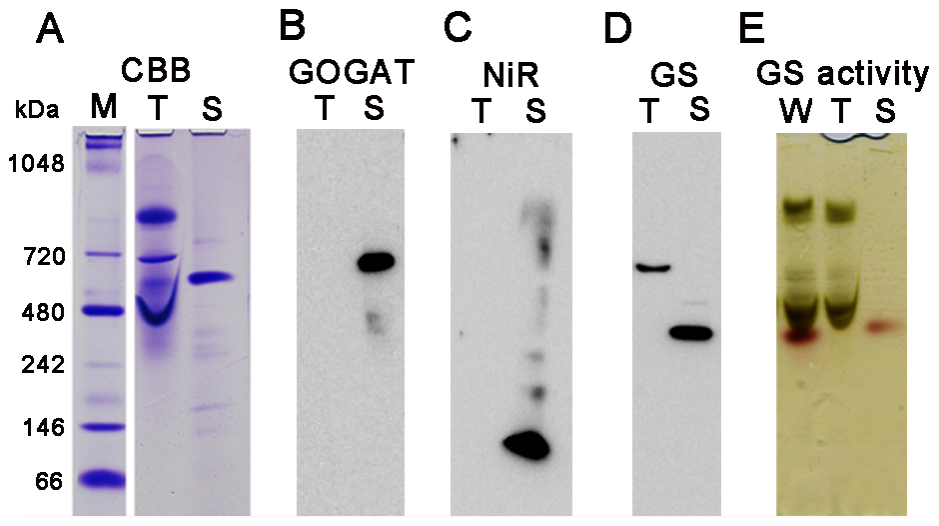
Protein complexes in spinach chloroplasts analyzed by using Native PAGE. CBB staining (A), western blots (B–D) and in-gel GS activity staining (E) after Native PAGE analysis of thylakoid (T) and stroma (S) proteins extracted from spinach chloroplasts. For in-gel GS activity assay, proteins extracted from whole chloroplasts (W) were also used. Lane M stands for the protein standard markers, but the migration distance does not necessarily correlate with the molecular mass in this Native PAGE. Samples loaded were derived from 10 µg (A–D) and 20 µg (E) on a chlorophyll basis of chloroplasts whose proteins were extracted.

Native PAGE without the CBB dye was performed for an in-gel GS activity assay because the dye interferes the activity staining in BN-PAGE. Native PAGE without detergents is limited to the separation of acidic proteins with a pI below the pH of the gel and is often characterized by protein aggregation and broadening of protein bands as compared with BN-PAGE. However, it is even milder than BN-PAGE and thought to offer advantages for retaining physiological protein assemblies and oligomerization. As shown in Figure 5A, the migration pattern of molecular weight markers (lane M) was very similar to that of BN-PAGE (lane M in Fig. 1A). Minor differences were seen in the pattern of stromal proteins with slower mobility of a major band (ribulose 1,5-bisphosphate carboxylase/oxygenase complex) compared to that in BN-PAGE (lane S in Figs. 1A&5A). In contrast, the pattern of thylakoid proteins was rather different between the two PAGEs especially for the slower mobility of chlorophyll binding protein complexes (around 200, 300 and 600 kDa in BN-PAGE; lane T in Figs. 1A&5A). For the patterns of the three nitrogen assimilatory enzymes, no large difference was seen except for the apparent lack of the 190 kDa GOGAT monomer, and instead, smear bands migrating around the 480 kDa marker were observed in addition to the major band around 720 kDa marker on Native PAGE (Fig. 5B). Thus, the behavior of the monomer form of GOGAT appears to be different between the two native PAGEs. For NiR (Fig. 5C), more intensive shifts, as compared to that on BN-PAGE (Fig. 1C), were observed even at neutral pH. These results may represent more physiological situation than those in BN-PAGE. As for GS (Fig. 5D), the distribution pattern was basically the same as those on BN-PAGE (Fig. 1D) except that the mobility of the band in the thylakoid fraction was slightly slower. The identity of a very faint band observed above the major band in stroma is not clear at present. Thus, multiple protein complexes common to any of the three nitrogen assimilation enzymes were not observed also by this Native PAGE analysis. Since the profile of the bands detected by the anti-GS antibody remained basically the same between the analyses of BN-PAGE and Native PAGE, an in-gel GS activity assay using Native PAGE was performed in order to evaluate whether the molecular species in each fraction are enzymatically active or not (Fig. 5E). After the run of the Native PAGE, chlorophyll-binding proteins were detected as a green color in the lanes of thylakoids (T) and whole chloroplasts (W). Subsequent GS activity staining caused yellowish background of the whole gel and reddish staining of only the 420 kDa protein in the lanes of stroma (lane S) and whole chloroplasts (lane W), in agreement with the results of the leaf GS activity staining of *M. medicago* plants described in the previous section. The GS transferase activity in solution was also measured to be 2.1 µmol min^−1^ mg^−1^ total protein in the stromal extract. The activity in the thylakoid extract was about 0.07 µmol min^−1^ mg^−1^ total protein, which was barely above the background level. Taken together, the 420 kDa GS holoenzyme present in stroma was shown to be a catalytically active form while the slower migrating protein complex (560 kDa) detected by the anti-GS antibody in thylakoids appeared to have no significant GS activity. The 560 kDa protein in thylakoids may represent an inactive form caused by a post-translational modification as described in the previous section. In this regard, the higher molecular weight form could result from the covalent binding of ubiquitin or ubiquitin-like proteins of approx. 10 kDa; Arabidopsis GS2 has been identified as a potential substrate for SUMO (small ubiquitin-like modifier) by the yeast two-hybrid system [27] although SUMOylation has not been tested in the plants. Another possibility has been postulated for the slower migrating form of the root nodule GS2 protein to be a non-processed precursor form which contains the transit peptide [25]. Analytical studies such as a proteomic analysis of the isolated protein are required to verify these possibilities. Reactivity of the anti-GS antibody we used was examined by additional western analyses (Figure S1 and S2), but we can't totally deny the possibility that the higher molecular weight forms (the 560 kDa protein and the 55 kDa polypeptide) detected by the GS antibody are a non-GS protein that may contain some antigenic structures common to GS proteins.

### Fd-binding ability of multiple forms of GOGAT and NiR proteins

**Figure 6:**
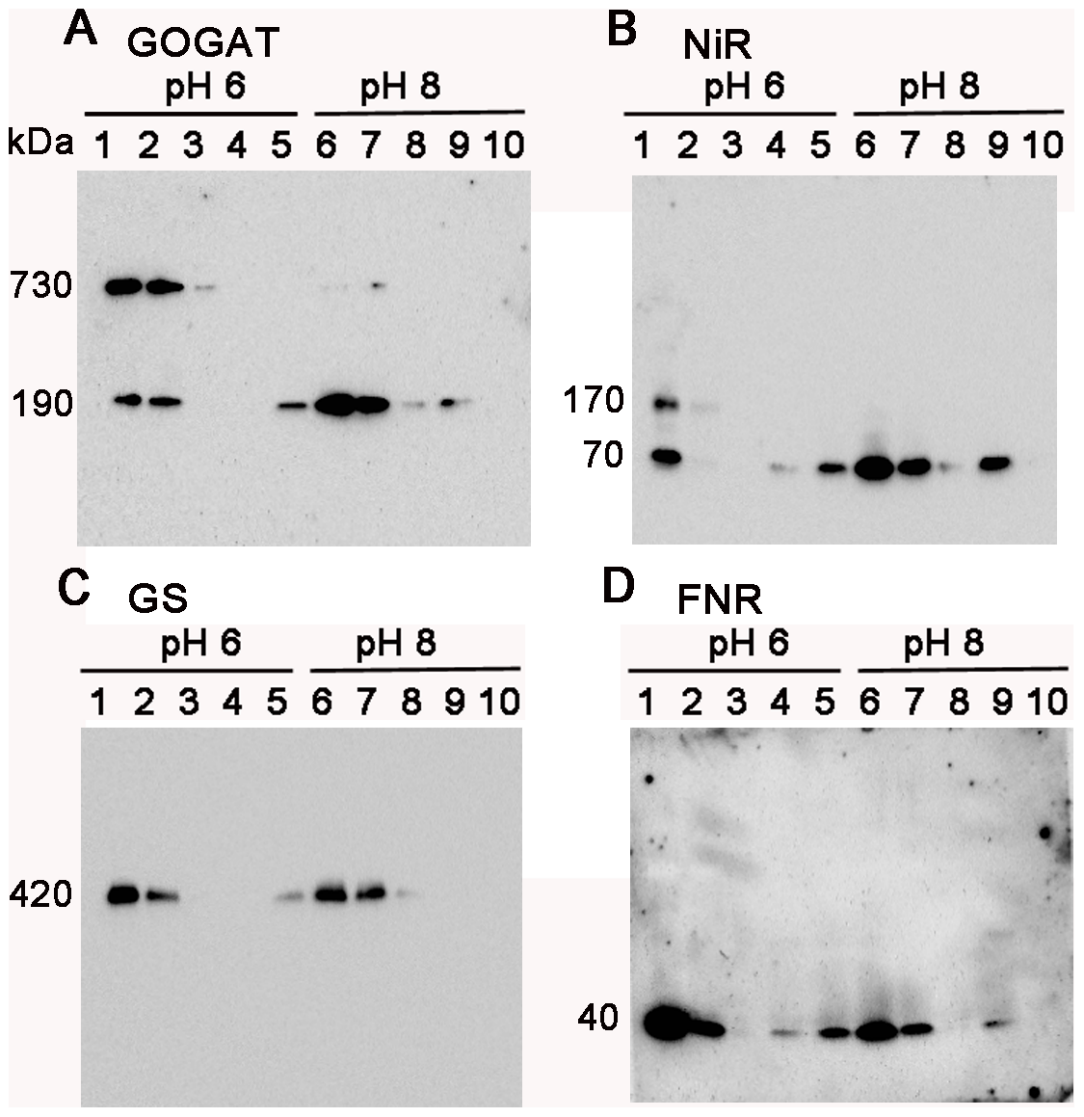
Fd-binding assay of protein complexes in the stromal fraction of spinach chloroplasts. Stromal proteins extracted from spinach chloroplasts by using potassium phosphate buffer at pH 6 (lane 1) or pH 8 (lane 6) were mixed with Fd resin, and the resulting unbound fractions (lanes 2&7), washed fraction (lanes 3&8) and fractions eluted with first (lanes 4&9) and second (lanes 5&10) one-column volume of the same buffer containing 0.2 M NaCl were collected and analyzed by western blots after BN-PAGE.

GOGAT and NiR are known to interact with Fd as a physiological electron donor. In order to evaluate the function of the multiple forms of GOGAT and NiR, the ability of each form for binding with Fd was investigated using the stromal extracts prepared under different pH conditions (Fig. 6). FNR as another major Fd-dependent enzyme and GS as a non-Fd dependent enzyme were also analyzed as controls. We routinely use an affinity resin with recombinant Fd from the cyanobacteria *L. boryana* [19] for the purification of Fd-dependent enzymes because of its stability on an affinity resin and because *L. boryana* Fd functions as an electron donor for Fd-dependent enzymes in higher plants to an extent comparable to that achieved by plant Fds. At pH 8, where GOGAT, NiR and FNR are mostly present as monomers in the stromal extracts, profiles of the Fd-binding and elution were quite similar among these three enzymes (lanes 6–10 in panels A, B and D); part of the enzymes were bound and released with first one-column volume of the elution buffer containing 0.2 M NaCl. The relative intensity of the signals in the elution fraction (lane 9) was somewhat higher for NiR than for FNR and GOGAT. This is consistent with the binding strength of these enzymes in the leaf extracts of maize and potato on Fd-affinity chromatography; in the order of GOGAT<FNR<NiR (T. Hase and Y. Chikuma, personal communications). In contrast, no detectable band was seen in the elution fraction of GS (lane 9 in panel C), indicating no significant binding. Thus, monomer forms of GOGAT and NiR, as well as FNR, at least partly bind with Fd in the stromal extracts under the conditions of pH 8. On the other hand, at pH 6, higher molecular weight forms of GOGAT (730 kDa) and NiR (170 kDa) in addition to the monomer forms were clearly observed in the extracts (lanes 1 in panels A and B). For NiR protein, the 170 kDa band together with the 70 kDa monomer band was greatly diminished in the unbound fraction (lane 2 in panel B), indicating their efficient binding with Fd. The reason for the detection of only the monomer band in the elution fraction (lanes 4,5 in panel B) is assumed to be due to the dissociation of the 170 kDa molecule under the conditions of 0.2 M NaCl (as implied from the results in lane 2 of Fig. 2B). FNR as a control also showed similar binding profile with NiR, although only the monomer was detected under this condition (lanes 1–5, panel D). On the other hand in GOGAT, significant reduction in the intensity of the bands was not observed in the unbound fraction (lane 2 in panel A), but partial binding was indicated from the band in the elution fraction (lane 5 in panel A). Because the 730 kDa molecule appears to dissociate into the 190 kDa monomer under 0.1 M NaCl condition (lane 2 in Fig. 2A), it is not clear which molecules, either or both of the 730 kDa and the 190 kDa proteins, actually bound with Fd. Under pH 6 conditions, GS protein also appears to partly bind with Fd resin (lane 5 in panel C); whether this is due to non-specific binding or transient complex formation of GS with other Fd-binding proteins is not clear at present.

Taken together, both the monomer (70 kDa) and the higher molecular weight complex (170 kDa) of NiR exhibited the binding ability with Fd; oligomerization of NiR may be related to its other function such as enzyme activity and protein stability. On the other hand in GOGAT, the monomer binds with Fd at least pH 8, and the 730 kDa complex appears to have either negligible or low Fd-binding ability under pH 6 conditions. As for Fd-GOGAT from *Synechococcus* sp. PCC 6301, there is a report that two protein bands appeared after non-denaturing PAGE of purified GOGAT proteins, both presenting GOGAT activity *in situ* using methyl viologen as a small artificial electron donor although the data were not shown [28]. Thus, the higher molecular weight complex of GOGAT in spinach chloroplasts may itself retain catalytic activity but may assume conformation which reduces the binding with Fd for the purpose to regulate the GOGAT reaction in the stromal fraction.

**Figure 7:**
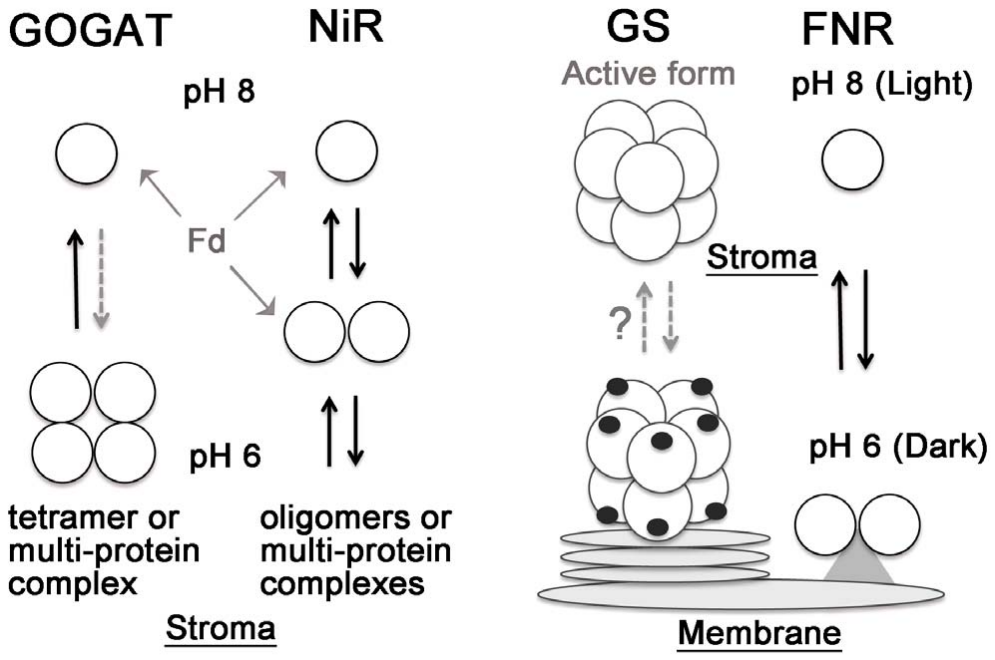
Models of pH-dependent complex formation and relocation of carbon- and nitrogen assimilatory enzymes in chloroplasts. Complex formation (possibly oligomerization) of GOGAT and NiR and localization of GS signals observed in this study are depicted together with a previously presented model for FNR [7]. GOGAT and NiR display pH-dependent conversion of monomers and oligomers (or protein complexes with unknown proteins) in the stromal fraction, which appears to be accompanied by differential binding to Fd (Fig. 6) and possibly by differential enzyme activity or protein stability. GS is postulated to exist both as an active enzyme of decamer in stroma and as a modified, probably inactive form (depicted with black dots) in the plastid membranes (such as thylakoids and envelop), which may interconvert by an unknown mechanism. FNR is known to relocate between stroma and thylakoids (possibly as a dimer for the stabilization of this enzyme) in the light, redox and pH-dependent manners [7], [8].

To sum up, individual complexes with larger molecular sizes of holoenzyme were detected for all the three nitrogen assimilatory enzymes, GOGAT, GS and NiR, studied in this article. Figure 7 shows schematic models for the pH-dependent conversion of monomers and oligomers (or multi-protein complexes) of GOGAT and NiR, and possible localization of GS inferred from this study, together with a previously presented model for FNR [7]. The pH-dependent conversion of monomers and multimeric complexes of GOGAT and NiR, which appears to be accompanied by differential binding to Fd and possibly by differential enzyme activity or protein stability, indicates the physiological significance of these complexes for the regulation of enzyme function in response to the conditions such as redox state in stroma and light availability. To our surprise, GS signal was observed as another inactive complex in thylakoid fraction (or some other plastid membranes) with a larger polypeptide, postulating a different regulatory mechanism from those of the above two enzymes, which involves different localization, similarly to the case of FNR, and possibly a novel post-translational modification.

## Supporting Information

Figure S1 **Western analysis of spinach proteins by SDS-PAGE (A) and BN-PAGE (B).** A: Total proteins from spinach leaves (lane L), whole chloroplasts (W), stroma (S) and thylakoid fraction (T) were analyzed by western blot after 7.5% SDS-PAGE probed with anti-GS antibody (GS) and preimune serum (PI). In spinach leaves, only GS2 isoprotein is considered to be expressed [ref. S1 in Figure S1] and is thought to be detected by the polyclonal antibody against maize GS1a proteins [ref. S2 in Figure S1] that shares 75% identity with spinach GS2, through the consequence of the cross-reactivity of the antibody by recognizing the epitopes common to the two GS isozymes. As expected from the western analysis of the whole chloroplasts by 2D BN/SDS-PAGE (Figure 1H), stroma and thylakoid fractions contained the polypeptides with distinct sizes of approx. 41 kDa and 51 kDa, respectively. The deduced MWs are somewhat different from those in the 2D analysis (45 kDa and 55 kDa), but the size in the stromal fraction of the 1D SDS-PAGE (41 kDa) appears to be closer to the estimated size of spinach GS2 protein (372 residues) and would be more accurate than the 2D analysis. B: Proteins extracted from whole chloroplasts were analyzed by western blot after BN-PAGE, probed with anti-GS antibody (GS) and preimune serum (PI). The numbers beside each band stand for the estimated molecular weights. All the samples loaded were derived from 10 µg on a chlorophyll basis of chloroplasts whose proteins were extracted.
(DOCX)

Figure S2 **Western analysis of maize proteins by BN-PAGE (A) and SDS-PAGE (B).** A: Thylakoid (T) and stroma (S) proteins were extracted from maize chloroplasts using the same protocol as described for spinach in the ‘Materials and Methods’ section, and were analyzed by western blot after BN-PAGE, probed with anti-GS antibody (GS). The pattern is quite similar to that of spinach leaves (Figure 1D), although the mobility of the major band detected in the lane of thylakoid proteins is slightly faster (approx. 540 kDa) than that of spinach (560 kDa). B: Total proteins from maize leaves (lane L), stroma (S) and thylakoid fraction (T) were analyzed by western blot after 7.5% SDS-PAGE, probed with anti-GS antibody (GS). The analysis showed the bands, 41 kDa for stroma and 49 kDa for thylakoids, which are similar to those of spinach (Figure S1A). The relative content of the maize GS2 protein in the stromal fraction appears to be lower compared to that of spinach. Additional lowest band detected in the lane of the whole leaves is thought to be a cytosolic GS1 protein (39 kDa), which is known to be absent in spinach leaves, leading to the confirmation that the anti-GS antibody reacts with both GS1 and GS2. Thus, the patterns of the GS signals detected in the stroma and thylakoid fractions of maize chloroplasts were basically the same as those of spinach on both BN-PAGE and SDS-PAGE analyses. The numbers beside each band stand for the estimated molecular weights.
(DOCX)
