# Supplementary material for: Multiple Complexes of Nitrogen Assimilatory Enzymes in Spinach Chloroplasts: Possible Mechanisms for the Regulation of Enzyme Function
Source: PLoS One. 2014 Oct 1;9(10):e108965. doi: 10.1371/journal.pone.0108965 (PMC4182809; doi:10.1371/journal.pone.0108965)
Supplement: Figure S1 — Western analysis of spinach proteins by SDS-PAGE (A) and BN-PAGE (B). A: Total proteins from spinach leaves (lane L), whole chloroplasts (W), stroma (S) and thylakoid fraction (T) were analyzed by western blot after 7.5% SDS-PAGE probed with anti-GS antibody (GS) and preimune serum (PI). In spinach leaves, only GS2 isoprotein is considered to be expressed [ref. S1 in Figure S1] and is thought to be detected by the polyclonal antibody against maize GS1a proteins [ref. S2 in Figure S1] that shares 75% identity with spinach GS2, through the consequence of the cross-reactivity of the antibody by recognizing the epitopes common to the two GS isozymes. As expected from the western analysis of the whole chloroplasts by 2D BN/SDS-PAGE (Figure 1H), stroma and thylakoid fractions contained the polypeptides with distinct sizes of approx. 41 kDa and 51 kDa, respectively. The deduced MWs are somewhat different from those in the 2D analysis (45 kDa and 55 kDa), but the size in the stromal fraction of the 1D SDS-PAGE (41 kDa) appears to be closer to the estimated size of spinach GS2 protein (372 residues) and would be more accurate than the 2D analysis. B: Proteins extracted from whole chloroplasts were analyzed by western blot after BN-PAGE, probed with anti-GS antibody (GS) and preimune serum (PI). The numbers beside each band stand for the estimated molecular weights. All the samples loaded were derived from 10 µg on a chlorophyll basis of chloroplasts whose proteins were extracted. (DOCX) [file pone.0108965.s001.docx]

**Supporting information S1**

**Multiple complexes of nitrogen assimilatory enzymes in spinach chloroplasts: possible mechanisms for the regulation of enzyme function.**

Yoko Kimata-Ariga*, Toshiharu Hase

Institute for Protein Research, Osaka University, 3-2 Yamadaoka, Suita, Osaka, 565-0871, Japan.

***Corresponding author**

E-mail: [a-yoko@protein.osaka-u.ac.jp](mailto:a-yoko@protein.osaka-u.ac.jp)

Address: Institute for Protein Research, Osaka University, 3-2 Yamadaoka, Suita, Osaka, 565-0871, Japan

Tel: +81-6-6879-8611, Fax: +81-6-6879-8613

**Figure S1**

**
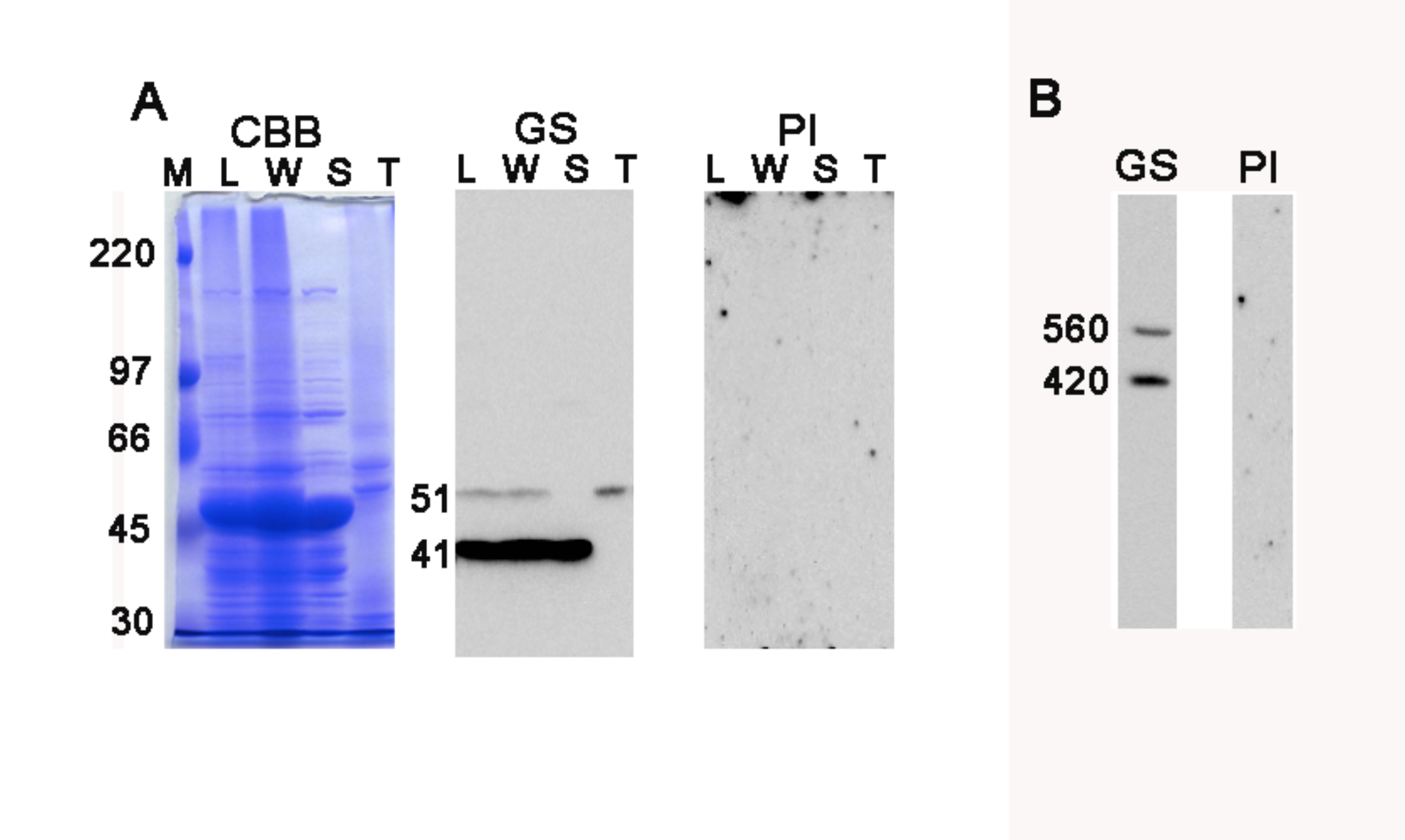
**

**Figure S1. Western analysis of spinach proteins by SDS-PAGE (A) and BN-PAGE (B)**. A: Total proteins from spinach leaves (lane L), whole chloroplasts (W), stroma (S) and thylakoid fraction (T) were analyzed by western blot after 7.5% SDS-PAGE probed with anti-GS antibody (GS) and preimune serum (PI). In spinach leaves, only GS2 isoprotein is considered to be expressed [ref. S1] and is thought to be detected by the polyclonal antibody against maize GS1a proteins [ref. S2] that shares 75% identity with spinach GS2, through the consequence of the cross-reactivity of the antibody by recognizing the epitopes common to the two GS isozymes. As expected from the western analysis of the whole chloroplasts by 2D BN/SDS-PAGE (Figure 1H), stroma and thylakoid fractions contained the polypeptides with distinct sizes of approx. 41 kDa and 51 kDa, respectively. The deduced MWs are somewhat different from those in the 2D analysis (45 kDa and 55kDa), but the size in the stromal fraction of the 1D SDS-PAGE (41 kDa) appears to be closer to the estimated size of spinach GS2 protein (372 residues) and would be more accurate than the 2D analysis. B: Proteins extracted from whole chloroplasts were analyzed by western blot after BN-PAGE, probed with anti-GS antibody (GS) and preimune serum (PI). The numbers beside each band stand for the estimated molecular weights. All the samples loaded were derived from 10 μg on a chlorophyll basis of chloroplasts whose proteins were extracted.

**Reference**

S1. McNally SF, Hirel B, Gadal P, Mann F, Stewart GR (1983) Glutamine synthetases of higher plants. Plant Physiol. 72: 22-25.

S2. Sakakibara H, Shimizu H, Hase T, Yamazaki Y, Takao T, Shimonishi Y, Sugiyama T (1996) Molecular identification and characterization of cytosolic isoforms of glutamine synthetase in maize roots. J Biol Chem. 271(47): 29561-8.
