# Supplementary material for: Multiple Complexes of Nitrogen Assimilatory Enzymes in Spinach Chloroplasts: Possible Mechanisms for the Regulation of Enzyme Function
Source: PLoS One. 2014 Oct 1;9(10):e108965. doi: 10.1371/journal.pone.0108965 (PMC4182809; doi:10.1371/journal.pone.0108965)
Supplement: Figure S2 — Western analysis of maize proteins by BN-PAGE (A) and SDS-PAGE (B). A: Thylakoid (T) and stroma (S) proteins were extracted from maize chloroplasts using the same protocol as described for spinach in the ‘Materials and Methods’ section, and were analyzed by western blot after BN-PAGE, probed with anti-GS antibody (GS). The pattern is quite similar to that of spinach leaves (Figure 1D), although the mobility of the major band detected in the lane of thylakoid proteins is slightly faster (approx. 540 kDa) than that of spinach (560 kDa). B: Total proteins from maize leaves (lane L), stroma (S) and thylakoid fraction (T) were analyzed by western blot after 7.5% SDS-PAGE, probed with anti-GS antibody (GS). The analysis showed the bands, 41 kDa for stroma and 49 kDa for thylakoids, which are similar to those of spinach (Figure S1A). The relative content of the maize GS2 protein in the stromal fraction appears to be lower compared to that of spinach. Additional lowest band detected in the lane of the whole leaves is thought to be a cytosolic GS1 protein (39 kDa), which is known to be absent in spinach leaves, leading to the confirmation that the anti-GS antibody reacts with both GS1 and GS2. Thus, the patterns of the GS signals detected in the stroma and thylakoid fractions of maize chloroplasts were basically the same as those of spinach on both BN-PAGE and SDS-PAGE analyses. The numbers beside each band stand for the estimated molecular weights. (DOCX) [file pone.0108965.s002.docx]

**Supporting information S2**

**Multiple complexes of nitrogen assimilatory enzymes in spinach chloroplasts: possible mechanisms for the regulation of enzyme function.**

Yoko Kimata-Ariga*, Toshiharu Hase

Institute for Protein Research, Osaka University, 3-2 Yamadaoka, Suita, Osaka, 565-0871, Japan.

***Corresponding author**

E-mail: [a-yoko@protein.osaka-u.ac.jp](mailto:a-yoko@protein.osaka-u.ac.jp)

Address: Institute for Protein Research, Osaka University, 3-2 Yamadaoka, Suita, Osaka, 565-0871, Japan

Tel: +81-6-6879-8611, Fax: +81-6-6879-8613

**Figure S2**

**
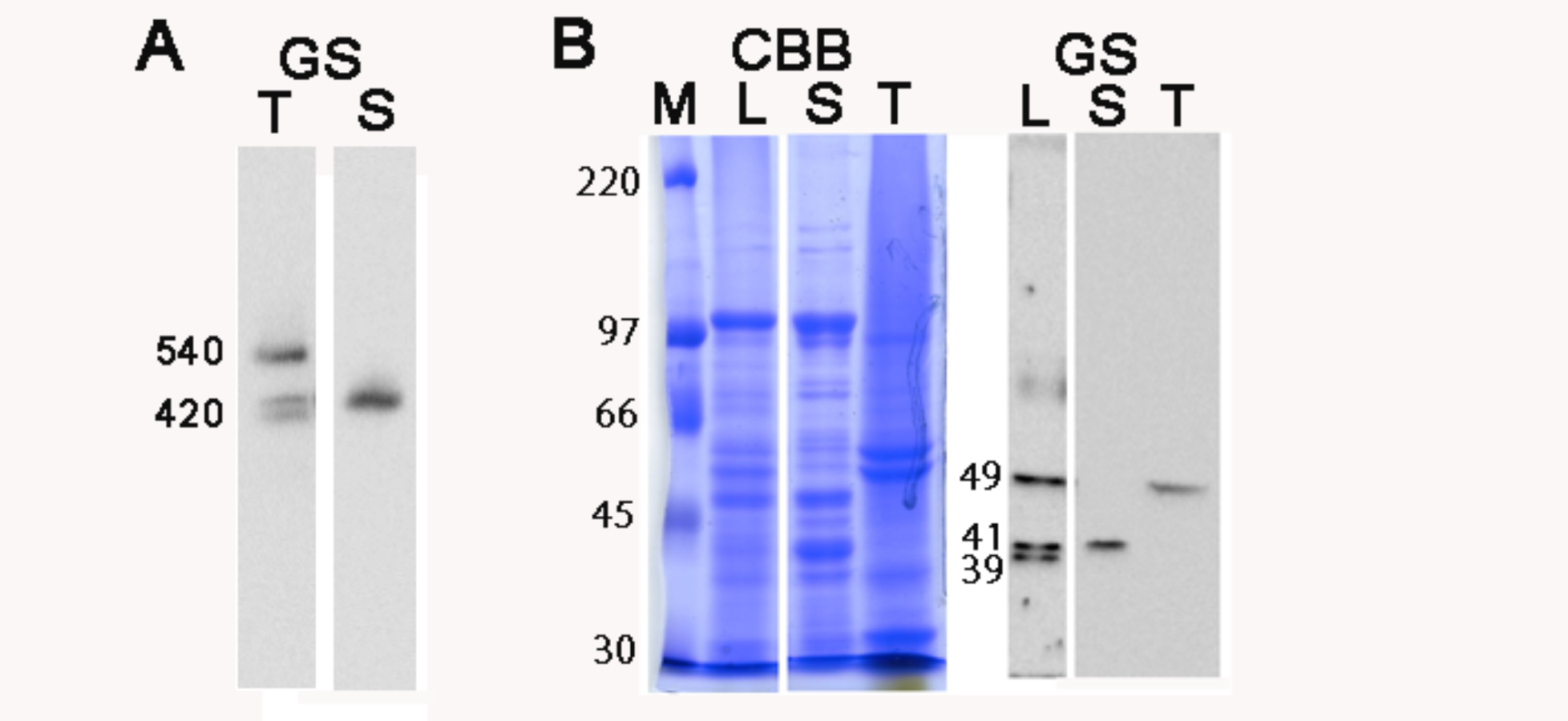
**

**Figure S2. Western analysis of maize proteins by BN-PAGE (A) and SDS-PAGE (B)**. A: Thylakoid (T) and stroma (S) proteins were extracted from maize chloroplasts using the same protocol as described for spinach in the ‘Materials and Methods’ section, and were analyzed by western blot after BN-PAGE, probed with anti-GS antibody (GS). The pattern is quite similar to that of spinach leaves (Figure 1D), although the mobility of the major band detected in the lane of thylakoid proteins is slightly faster (approx. 540 kDa) than that of spinach (560 kDa). B: Total proteins from maize leaves (lane L), stroma (S) and thylakoid fraction (T) were analyzed by western blot after 7.5% SDS-PAGE, probed with anti-GS antibody (GS). The analysis showed the bands, 41 kDa for stroma and 49 kDa for thylakoids, which are similar to those of spinach (Figure S1A). The relative content of the maize GS2 protein in the stromal fraction appears to be lower compared to that of spinach. Additional lowest band detected in the lane of the whole leaves is thought to be a cytosolic GS1 protein (39 kDa), which is known to be absent in spinach leaves, leading to the confirmation that the anti-GS antibody reacts with both GS1 and GS2. Thus, the patterns of the GS signals detected in the stroma and thylakoid fractions of maize chloroplasts were basically the same as those of spinach on both BN-PAGE and SDS-PAGE analyses. The numbers beside each band stand for the estimated molecular weights.
